# NSAIDs Alter Phosphorylated Forms of AQP2 in the Inner Medullary Tip

**DOI:** 10.1371/journal.pone.0141714

**Published:** 2015-10-30

**Authors:** Huiwen Ren, Baoxue Yang, Patrick A. Molina, Jeff M. Sands, Janet D. Klein

**Affiliations:** 1 Department of Medicine, Renal Division, Emory University School of Medicine, Atlanta, Georgia, United States of America; 2 Department of Physiology, Emory University, Atlanta, Georgia, United States of America; 3 Department of Pharmacology, School of Basic Medical Sciences, Peking University, Beijing, China; University of Bari Aldo Moro, ITALY

## Abstract

Vasopressin increases urine concentration through activation of aquaporin-2 (AQP2) in the collecting duct. Nonsteroidal anti-inflammatory drugs (NSAIDs) block prostaglandin E2 synthesis, and may suppress AQP2 producing a urine concentrating defect. There are four serines in AQP2 that are phosphorylated by vasopressin. To determine if chronic use of NSAIDs changes AQP2’s phosphorylation at any of these residues, the effects of a non-selective NSAID, ibuprofen, and a COX-2-selective NSAID, meloxicam, were investigated. Daily ibuprofen or meloxicam increased the urine output and decreased the urine osmolality significantly by days 7 through 14. Concomitantly, meloxicam significantly reduced total AQP2 protein abundance in inner medulla (IM) tip to 64% of control and base to 63%, respectively. Ibuprofen significantly decreased total AQP2 in IM tip to 70% of control, with no change in base. Meloxicam significantly increased the ratios of p^256^-AQP2 and p^261^-AQP2 to total AQP2 in IM tip (to 44% and 40%, respectively). Ibuprofen increased the ratio of p^256^-AQP2 to total AQP2 in IM tip but did not affect p^261^-AQP2/total AQP2 in tip or base. Both ibuprofen and meloxicam increased p^264^-AQP2 and p^269^-AQP2 ratios in both tip and base. Ibuprofen increased UT-A1 levels in IM tip, but not in base. We conclude that NSAIDs reduce AQP2 abundance, contributing to decreased urine concentrating ability. They also increase some phosphorylated forms of AQP2. These changes may partially compensate for the decrease in AQP2 abundance, thereby lessening the decrease in urine osmolality.

## Introduction

Cyclooxygenase (COX) enzymes can be blocked by nonsteroidal anti-inflammatory drugs (NSAIDs) and this leads to inhibition of prostaglandin (PG) synthesis and their analgesic and antipyretic responses. Treatment with high doses on a regular basis can have a marked anti-inflammatory effect. COX-1 is expressed constitutively and offers rapid responses to physiological challenges [1]. However, COX-2 is always induced by biological factors, such as TNF-alpha, IL-1, and IL-4 [2,3]. The renal side effects of NSAIDs are considered one of the major issues with the use of these important drugs. The other two most common sites of serious side effects are in the gastrointestinal [4,5] and cardiovascular systems [6–8]. Despite the presence of serious side effects, NSAIDs have proven to be valuable medications in clinical practice. Although always a possibility, renal side effects of NSAIDs are most likely to appear in patients with risk factors, such as renal dysfunction or reduced renal perfusion [5]. NSAIDs in a setting of renal dysfunction can lead to hyperkalemia, sodium retention, acute renal failure, reduced glomerular filtration rate, renal papillary necrosis and edema [6,9]. In patients with normal kidney function as well as those with renal dysfunction, the prolonged use of NSAIDS can cause a urine concentrating defect [10].

The antidiuretic response evoked by vasopressin binding to the vasopressin type 2 receptor (V2R) requires activation of aquaporin-2 (AQP2) trafficking to, and retention in, the apical plasma membrane in the collecting duct [11]. Vasopressin promotes water permeability by activation of cAMP and phosphorylation of AQP2 at Ser 256, Ser 261, Ser 264 and Ser 269, which then induces the translocation of AQP2 to the apical membrane [12–15]. Both Ser 256 and Ser 261 are involved in AQP2 accumulation in the apical plasma membrane [16]. Phospho-Ser 261 has been linked to ubiquitination and intracellular vesicle association of AQP2. Vasopressin and elevated cAMP levels result in increased initial phosphorylation of AQP2 at Ser 256, followed by increased Ser 264 and Ser 269 phosphorylation and reduced Ser 261 phosphorylation [16–18]. Phosphorylation at Ser 269 is important for AQP2 retention in the apical plasma membrane. These post translational modifications are essential for the steady-state redistribution of AQP2 between vesicles and the apical plasma membrane regulated by exocytosis and endocytosis [19].

There may be multiple effects of NSAIDs on AQP2 that result in a urine concentrating defect including decreasing the protein abundance and altering the channel activity [20]. In this study, we examined the role of chronic administration of two different NSAIDs on urine concentration and on the abundance of AQP2 and the four phospho-AQP2s.

## Materials and Methods

### Animals

Sprague-Dawley (SD) rats (Charles River Laboratories, Wilmington, MA) were maintained in accordance with the National Institutes of Health Guide for the Care and Use of Laboratory Animals. Animal care was supervised by the Emory University Division of Animal Research. The animals were euthanized by decapitation. These studies were approved by the Emory University Institutional Animal Care and Use Committee (protocol number 2002941).

### Metabolic cage studies

100 mg/kg meloxicam or 5 mg/kg ibuprofen dissolved in gelatin (JELL-O) were fed to male rats daily. Pure gelatin was used as a vehicle. On days 7 and 14, rats were placed in metabolic cages (Harvard Apparatus, Holliston, MA) for 24 hr urine collection. Trunk blood was collected upon decapitation and serum separated for analysis of: creatinine, urea, and vasopressin levels. Creatinine was measured using the QuantiChrom Creatinine Assay kit (BioAssay Systems, Hayward, CA) according to the manufacturer’s instructions. Urea was measured using the colorimetric Infinity reagent (Thermo Fisher Scientific, Norcross, GA) according to the manufacturer’s instructions. Vasopressin levels were measured by ELISA following manufacturer’s instructions (Enzo Life Sciences, Farmingdale, NY). Urine volume was determined using weight, assuming a density of 1 g/ml. Urine osmolality was measured using a Wescor vapor pressure osmometer 5520 (Logan, UT). Urine PGE2 was measured by ELISA using the Parameter assay kit (R and D Systems, Minneapolis, MN) according to the manufacturer’s instructions. Creatinine clearance was calculated from the plasma and urine creatinine values and urine volume.

### Protein Analysis

Rat kidneys were dissected into inner medullary tip and base. Using the distance from the inner-outer medullary border to the tip, we take the distal 40% as tip and the remainder as base. Tissue was placed into ice-cold isolation buffer (10 mM triethanolamine, 250 mM sucrose, pH 7.6, 1 μg/ml leupeptin, and 2 mg/ml PMSF), homogenized, then SDS was added to a final concentration of 1% for Western analysis of the total cell lysate. Total protein in each sample was measured by a modified Lowry method (Bio-Rad DC protein assay reagent, Bio-Rad, Richmond, CA). Proteins were size-separated by SDS-PAGE on Laemmli gels, then electroblotted to PVDF membranes. PVDF membranes were blocked for 60 min with 5% of blotting grade nonfat dry milk (Bio-Rad, Hercules, CA) before overnight incubation with primary antibodies: our total AQP2 and UT-A1, p^256^-AQP2 (Biorbyt, Burlington, NC), p^261^-AQP2 (Avivasysbio, San Diego, CA), p^264^-AQP2 (Thermo Fisher Scientific), and p^269^-AQP2 (Thermo Fisher Scientific). All phospho-AQP2 primary antibodies were used at a concentration of 1:1000, and total AQP2 primary antibody at 1:4000. Attached primary antibodies were identified using Alexa Fluor 680-linked anti-rabbit IgG (Molecular Probes, Eugene, OR) and visualized using infrared detection with the LICOR Odyssey protein analysis system. We performed densitometry using all of the AQP2 bands between 35 and 50 kDa, plus the 29 kDa band. To verify equal loading of the gels, we performed total protein staining of the blots using Ponceau S, followed by densitometry. We normalized each protein of interest to the loading control.

### Immunohistochemistry

Mice kidneys were perfused and paraformaldehyde fixed, and paraffin embedded as described previously [21]. Paraffin sections of 4 μm thickness were sliced. Sections were de-waxed and hydrated in preparation for immunostaining as previously described [22]. Sections were incubated overnight at 4°C with the AQP2 primary antibodies at 1:10,000. Slides were washed free of primary antibody and then incubated for 2 hours in peroxidase-conjugated secondary antibody (donkey anti-rabbit IgG). DAB (diaminobenzidine) and 35% H_2_O_2_ were added to detect peroxidase activity identifying the primary antibody. Slides were also stained with Mayers Hematoxylin to visualize nuclei. Stained sections were visualized using a bright field on an Olympus IX71 inverted microscope. IMCDs were imaged at with a 40X objective.

### Statistics

Data were analyzed using a paired Student’s t-test or repeated measures ANOVA, followed by Fisher’s least significant difference analysis for multiple comparisons. *P*<0.05 was considered statistically significant. Data are presented as mean ± SEM.

## Results

### Urine output and urine osmolality in rats treated by meloxicam and ibuprofen

**Fig 1:**
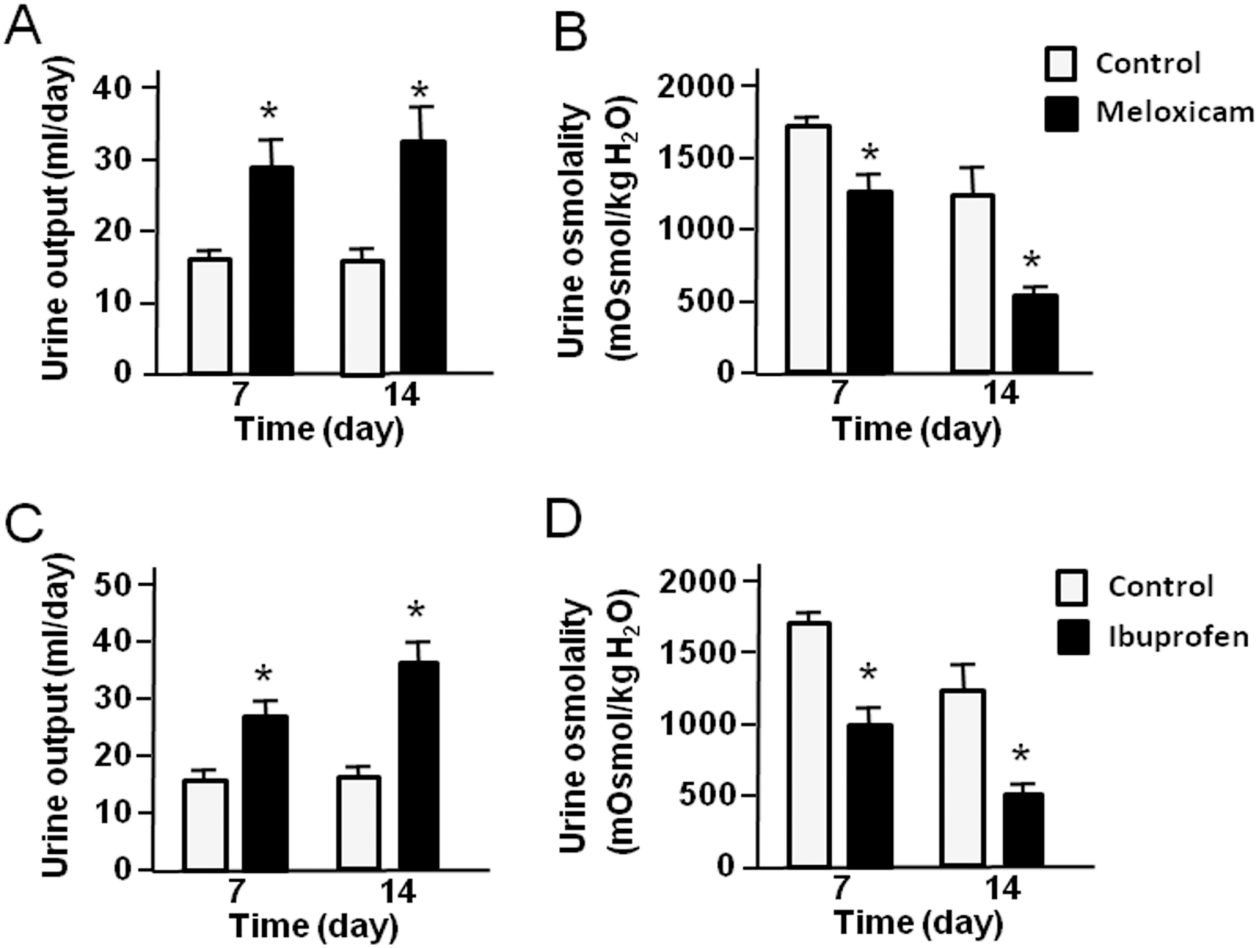
Urine output and urine osmolality in rats treated with meloxicam or ibuprofen. (A-B) the effect of meloxicam on urinary concentrating ability. Rats were fed with (black bars) or without (white bars) 100 mg/kg meloxicam daily and 24 hour urines collected on days 7 and 14: (A) urine output, (B) urine osmolality. (C-D) the effect of ibuprofen on urinary concentrating ability. Rats were fed with (black) or without (white) 5 mg/kg ibuprofen daily and 24 hour urines collected on days 7 and 14: (C) urine output, (D) urine osmolality. Bars = Mean ± SEM, n = 12 rats per group; *P<0.05 compared with control rats.

To investigate the effect of meloxicam and ibuprofen on urinary concentrating ability, rats were treated with meloxicam (100 mg/kg) and ibuprofen (5 mg/kg) every day for 14 days and 24 hour urine was collected on days 7 and 14. As shown in Fig 1A and 1B, urine output of the meloxicam-treated rats was significantly increased compared with control rats after 7 days and 14 days of administration. Likewise, treatment with meloxicam resulted in a significant decrease in urine osmolality compared with levels in control rats. The increase after 7 days was maintained over the subsequent 7 days. Similar results were revealed in ibuprofen-treated rats (Fig 1C and 1D), ie., urine output was significantly increased and urine osmolality was significant decreased compared with the controls after 7 days and 14 days of treatment, indicating that both meloxicam and ibuprofen produce a urine concentration defect in rats. There was no difference in plasma creatinine or urea between control, meloxicam, and ibuprofen treated animals. There was also no difference in creatinine clearance in control or treated animals (Table 1). Urine PGE2 was significantly reduced in the ibuprofen treated rats; meloxicam also reduced it but the decrease was not statistically significant (Table 1).

**Table 1:** Blood and urinary chemistry in control, meloxicam or ibuprofen treated rats on day 14.

	Control (n = 11)	Meloxicam (n = 12)	Ibuprofen (n = 12)
Original Body weight, g	183.3 ± 2.8	192.3 ± 6.3	193.0 ± 6.3
Final Body weight, g	255.5 ± 2.4	299.8 ± 6.4*	302.5 ± 6.4*
Urine output, ml per 24 h	15.0 ± 2.1	33.7 ± 5.1*	32.8 ± 3.2*
Urine osmolality, mOsm/kg H_2_O	1421 ± 319	475 ± 81*	578 ± 121*
Urinary Na, mM	101.8 ± 5.3	100.9 ± 10.7	108.3 ± 21.7
Urinary K, mM	174.3 ± 8.5	157.4 ± 17.1	208.5 ± 42.3
Urinary Cl, mM	126.2 ± 8.9	105.7 ± 14.1	142.1 ± 35.4
Urinary creatinine, mM	3.1 ± 0.9	3.4 ± 1.0	2.6 ± 0.9
Urinary excretion of Na, mmol/kg/day	8.2 ± 0.6	10.6 ± 1.9	15.3 ± 4.7
Urinary excretion of K, mmol/kg/day	14.0 ± 1.0	14.7 ± 1.8	29.4 ± 9.1
Urinary excretion of Cl, mmol/kg/day	10.1 ± 1.1	9.5 ± 1.4	20.2 ± 7.8
Urinary excretion of urea, mmol/kg/day	24.1 ± 0.6	30.5 ± 1.7*	35.1 ± 3.2*
Urinary excretion of creatinine, mmol/kg/day	0.33 ± 0.05	0.36 ± 0.1	0.34 ±0.1
Creatinine clearance, ml/min	0.22 ± 0.05	0.26 ± 0.08	0.25 ± 0.07
Plasma urea, mM**	7.3 ± 0.2	7.3 ± 0.2	7.7 ± 0.7
Plasma creatinine, μM**	288.1± 16.7	286 ± 15.7	265 ± 13.8
Urine PGE2, μM	6.1 ±1.8	3.1 ± 1.1	1.3 ± 0.2*
Plasma vasopressin, pg/ml**	0.60 ± 0.06	0.70 ± 0.04	0.70 ± 0.04

Data are presented as mean ± SEM,

*P<0.05 compared with control rats,

**n = 6 for plasma measurements

### Effect of meloxicam and ibuprofen on AQP2 abundance in IM tip and base

**Fig 2:**
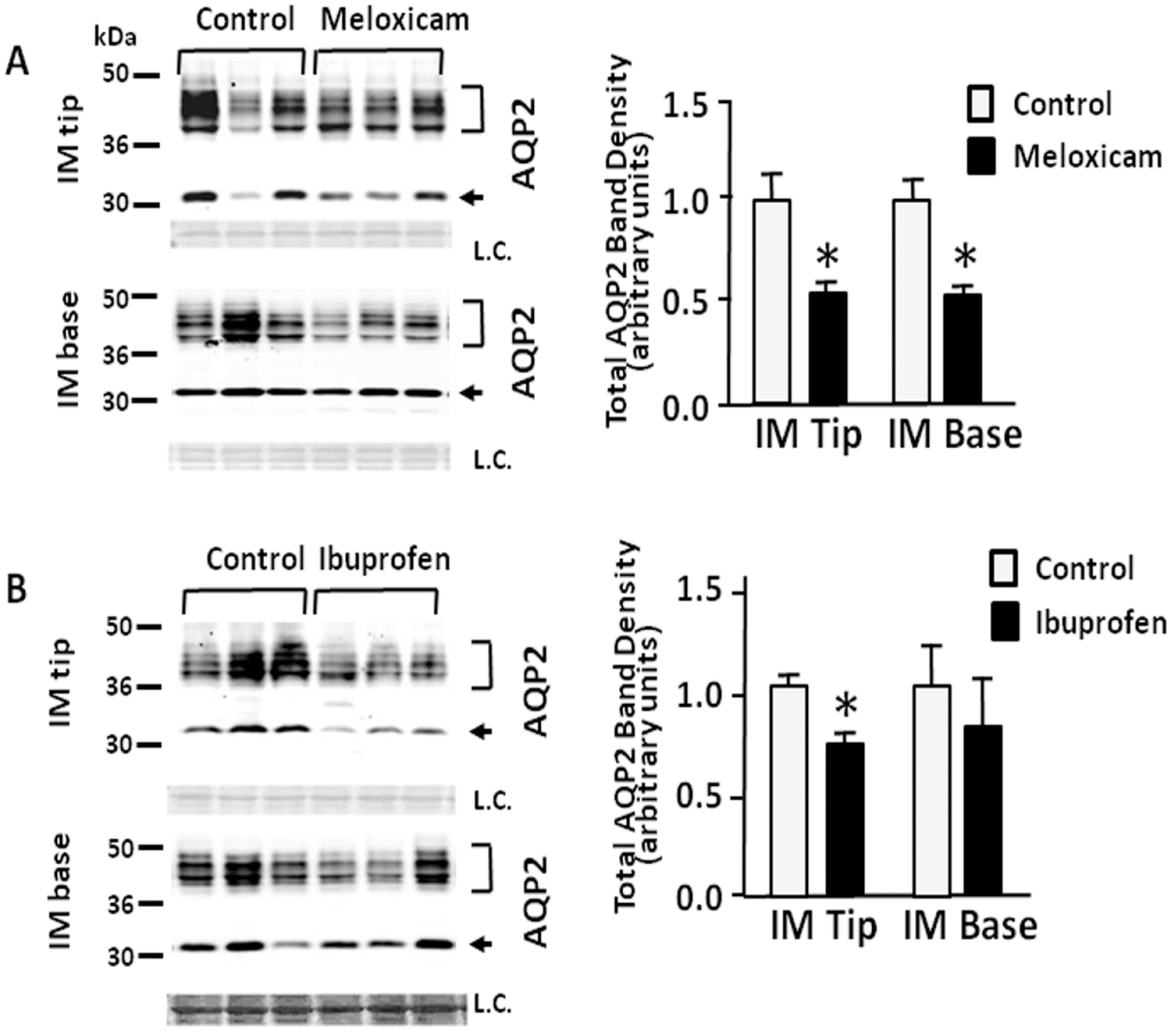
Effect of meloxicam and ibuprofen on AQP2 abundance in inner medullary tip and base. Shown are representative Western blots (left hand panels) from IM tips or bases from rats after 14 days treatment with or without meloxicam (top) or ibuprofen (bottom) probed for total AQP2 (arrows) The top bracket shows 35–50 kDa glycosylated forms; the bottom arrow designates the 29 kDa unglycosylated AQP2. L.C. = loading control. The right hand panels provide combined densitometry from n = 12 rats per group. Bars = Mean ± SEM; *P<0.05 compared with control rats.

**Fig 3:**
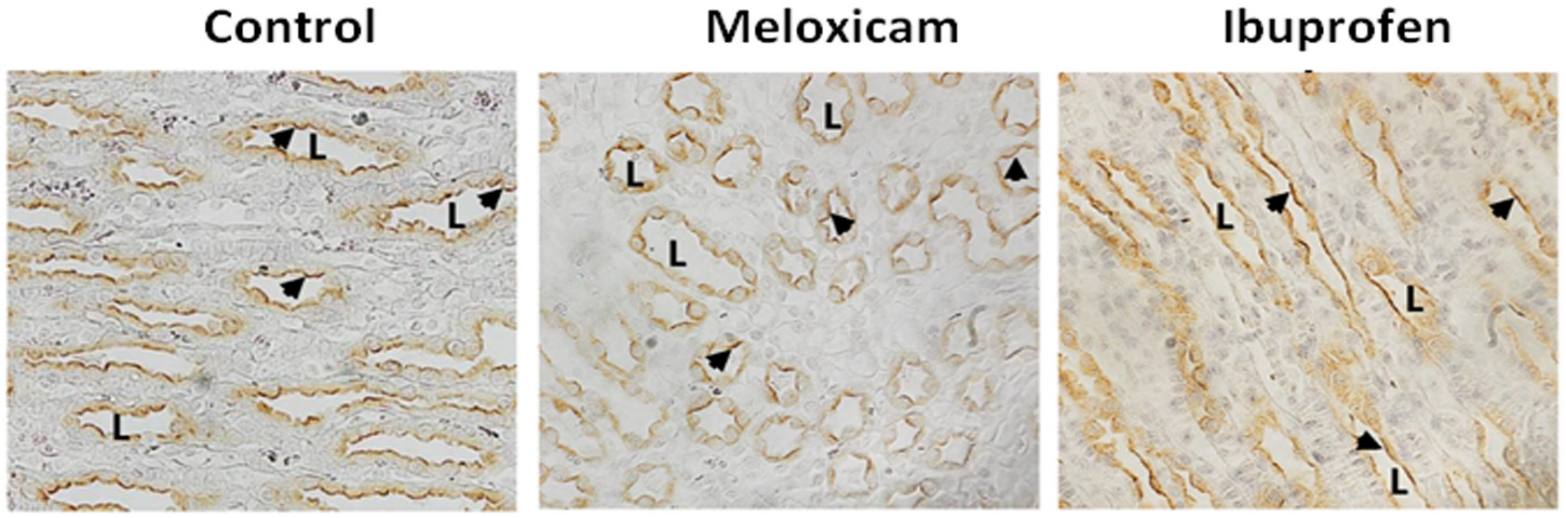
Immunohistology of kidneys showing the cellular location of AQP2. Perfusion fixed kidneys from control, meloxicam- and ibuprofen-treated mice were prepared for histology examination as described in the Methods section. AQP2 is denoted by the brown stain indicating positive peroxidase product. Slides were counterstained with hematoxylin to show nuclei. L indicates examples of the lumen of the collecting ducts. Arrows show the apical staining location of the AQP2. Magnification, 400x.

The expression of AQP2 protein was determined in IM tip and base. The AQP2 antibody recognizes both the unglycosylated 29-kDa and the 35- to 50-kDa glycosylated bands in both IM tip and base. Fig 2A shows representative Western blots of AQP2 from rats treated with the two NSAIDs. After the treatment with meloxicam (100 mg/kg), the total AQP2 expression was significantly reduced to 64% and 63% of control levels in IM tip and base, respectively (Fig 2A). In contrast, while ibuprofen decreased the AQP2 level to 70% in IM tip; there was no change in the levels in the IM base of the treated animals. By immunohistochemistry, there was no obvious difference in AQP2 localization between control and either meloxicam or ibuprofen treated animals (Fig 3).

### Effect of meloxicam on the phosphorylated forms of AQP2

**Fig 4:**
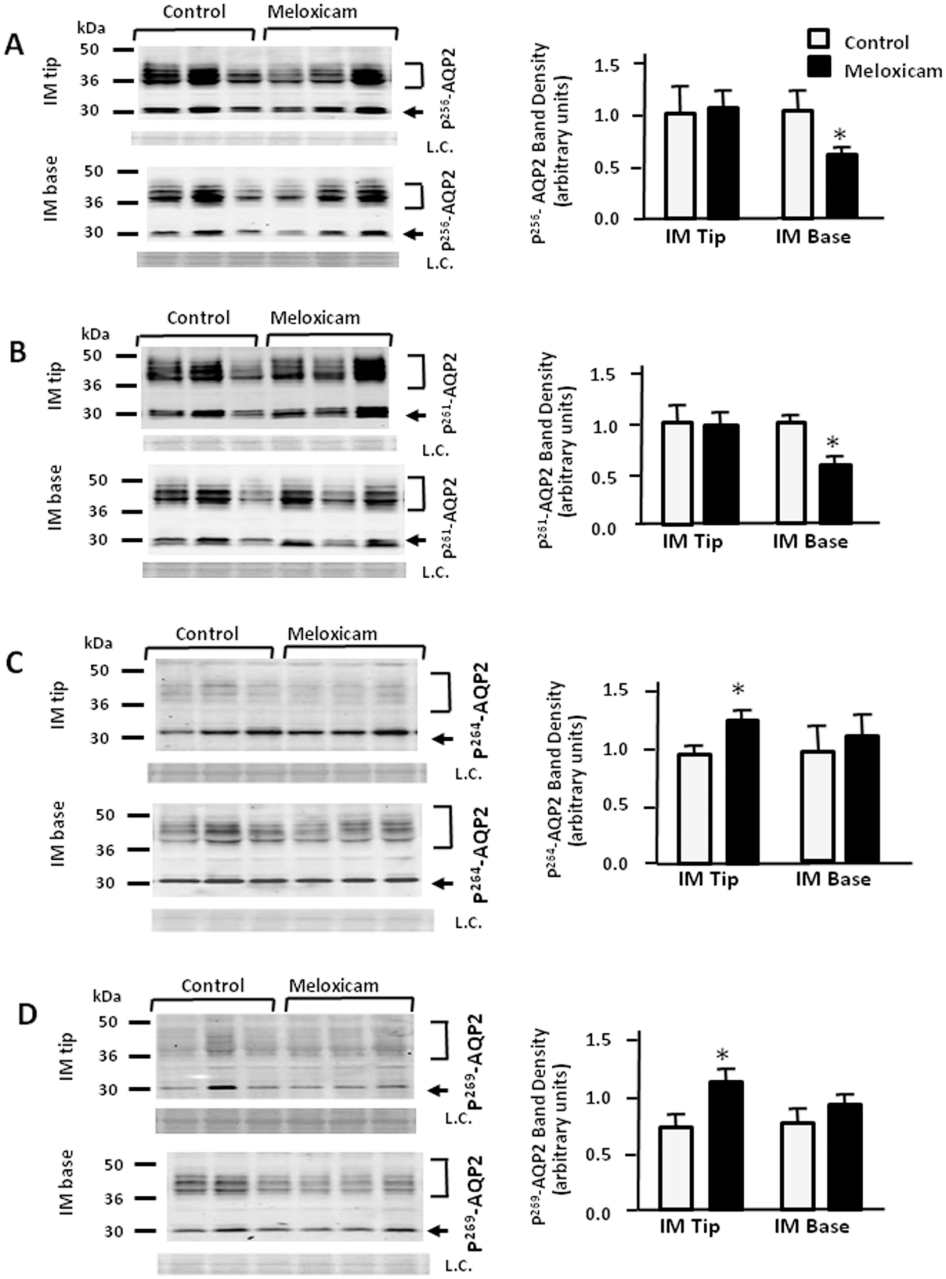
Effect of meloxicam on the phosphorylated forms of AQP2. Tissue samples were isolated from rat IM tip and base after 14 days treatment without or with meloxicam (100 mg/kg) and analyzed by Western blot for phosphorylated AQP2 (arrows) as follows: (A) p256AQP2, (B) p261AQP2, (C) p264AQP2 and (D) p269AQP2. Left panels provide representative Western blots. The top bracket shows 35–50 kDa glycosylated forms; the bottom arrow designates the 29 kDa unglycosylated AQP2. L.C. = loading control. Right bar graphs show phosphoAQP2 combined densitometry from n = 12 rats per group. All proteins were normalized to loading controls. White bars, control; black bars, meloxicam treated. Bars = Mean ± SEM, n = 12 rats per group; *P<0.05 compared with control rats.

**Fig 5:**
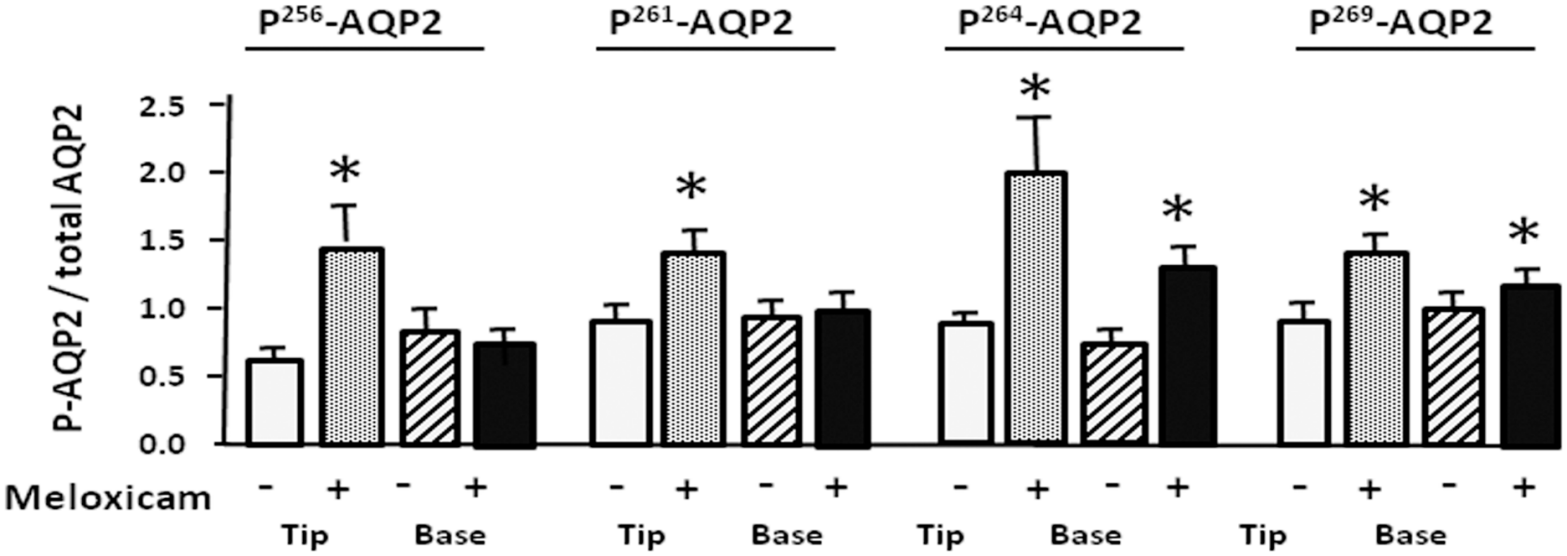
Effect of meloxicam on the phosphorylated forms of AQP2 to total AQP2. Bar graphs show the ratio of p256AQP2, p261AQP2, p264AQP2 and p269AQP2 to total AQP2. White bars, control in IM tip; gray bars, meloxicam treated in IM tip; white and oblique line bars, control in IM base; gray and oblique line bars, meloxicam treated in IM base. All proteins were normalized to loading controls. Bars = Mean ± SEM, n = 12 rats per group; *P<0.05 compared with control rats.

To investigate AQP2 phosphorylation in rat IM tip and base we used phospho-specific antibodies to AQP2 phosphorylated at Ser 256, Ser 261, Ser 264 and Ser 269. These phosphorylated proteins were assessed in meloxicam-treated rats. Meloxicam attenuated p^256^-AQP2 and p^261^-AQP2 expression to 68% and 62% of control levels in IM base, but not in IM tip (Fig 4A and 4B). Fig 4C and 4D show that p^264^-AQP2 and p^269^-AQP2 expression in control rats was attenuated by more than 23% and 48% in IM tip exposed to meloxicam. The ratio of phosphorylated AQP2 to total AQP2 was determined and is presented in bar graph form in Fig 5. The ratios of p^256^-AQP2 and p^261^-AQP2 to total AQP2 were preserved in the IM base while the phosphorylated forms in the IM tip were markedly increased by 44% and 40% compared with control rats (Fig 5A and 5B). The ratios of p^264^-AQP2 and p^269^-AQP2 to total AQP2 almost doubled in the IM tip, and showed a more modest but statistically significant increase (25% and 40%, respectively) in IM base (Fig 5).

### Effect of ibuprofen on the phosphorylated forms of AQP2

**Fig 6:**
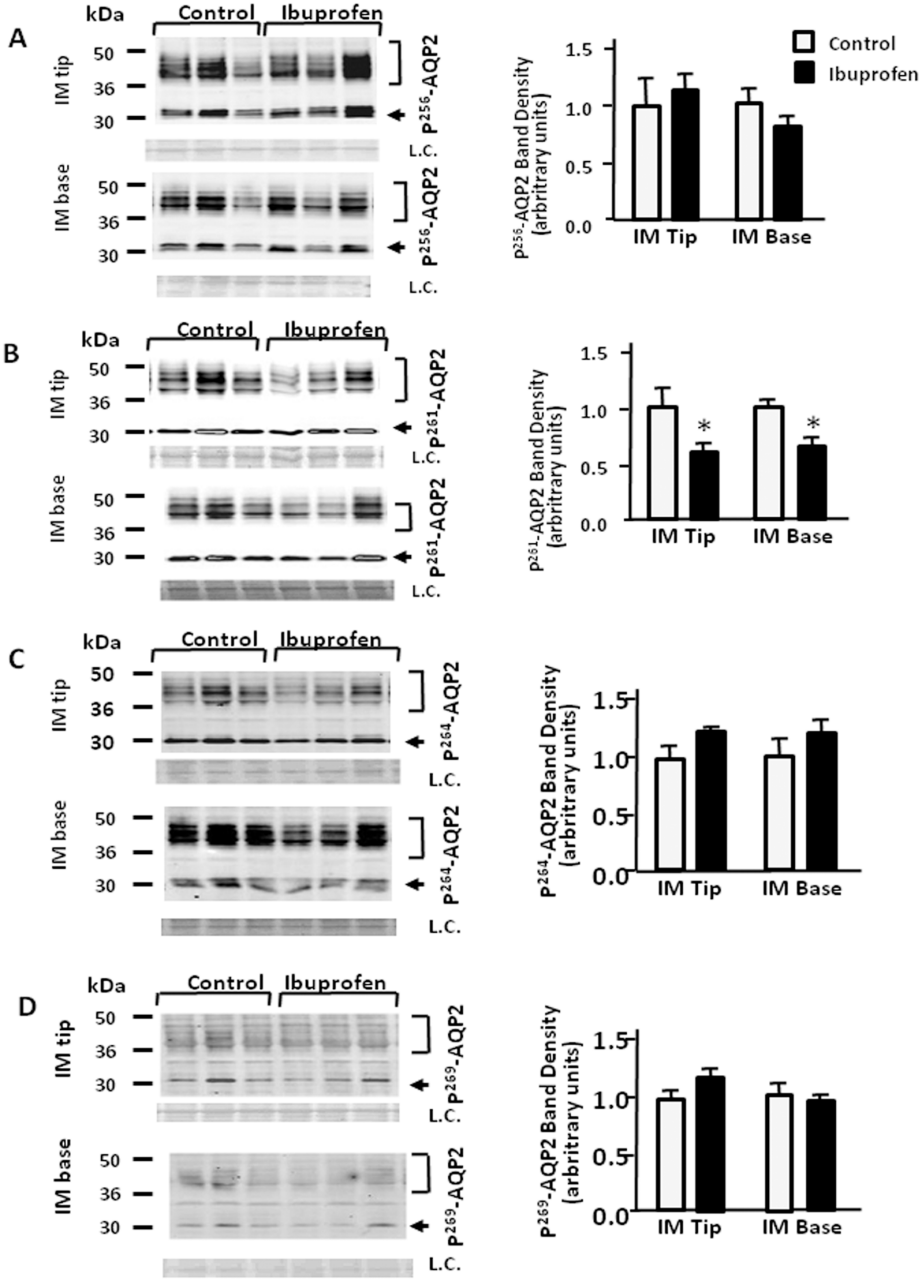
Effect of ibuprofen on the phosphorylated forms of AQP2. Tissue samples were isolated from rat IM tip and base after 14 days treatment without or with ibuprofen (5 mg/kg). and analyzed by Western blot for phosphorylated AQP2 (arrows) as follows: (A) p256AQP2, (B) p261AQP2, (C) p264AQP2 and (D) p269AQP2. Left panels provide representative Western blots. The top bracket shows 35–50 kDa glycosylated forms; the bottom arrow designates the 29 kDa unglycosylated AQP2. L.C. = loading control. Right bar graphs show phosphoAQP2 combined densitometry from n = 12 rats per group. All proteins were normalized to loading controls. White bars, control; black bars, ibuprofen treated. Bars = Mean ± SEM; *P<0.05 compared with control rats.

**Fig 7:**
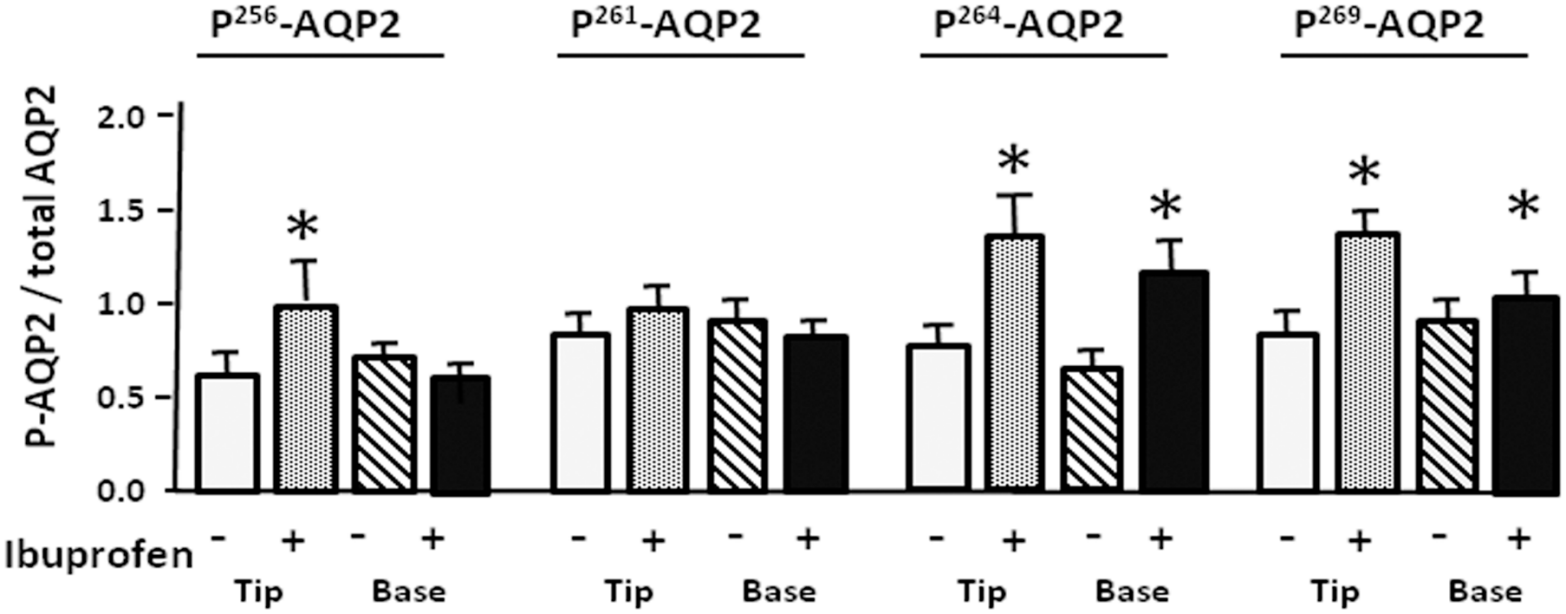
Effect of ibuprofen on the phosphorylated forms of AQP2 to total AQP2. Bar graphs show the ratio of p256AQP2, p261AQP2, p264AQP2 and p269AQP2 to total AQP2. White bars, control in IM tip; gray bars, ibuprofen treated in IM tip; white and oblique line bars, control in IM base; gray and oblique line bars, ibuprofen treated in IM base. All proteins were normalized to loading controls. Bars = Mean ± SEM, n = 12 rats per group; *P<0.05 compared with control rats.

After the treatment with ibuprofen, p^261^-AQP2 was significantly decreased to 71% and 68% of control levels in IM tip and base, respectively (Fig 6B). Ibuprofen had no effect on p^256^-AQP2, p^264^-AQP2 and p^269^-AQP2 in IM tip and base (Fig 6A, 6C and 6D). Despite the 15% increase in the ratio of p^256^-AQP2 to total AQP2 in the IM tip in response to ibuprofen, no significant differences were revealed in the expression of p^256^-AQP2 in IM base or p^261^-AQP2 in IM tip or base (Fig 7A and 7B). As shown in Fig 6C and 6D, ibuprofen significantly increased the ratios of p^264^-AQP2 and p^269^-AQP2 to total AQP2 in IM tip (p^264^-AQP2: to 43%; p^269^-AQP2: to 46% above control levels) and base (p^264^-AQP2: to 21%; p^269^-AQP2: to 13% above control levels). The degree of increase resulting from ibuprofen treatment was mild compared with the response to meloxicam treatment.

### UT-A1 abundance response to meloxicam and ibuprofen in rat IM tip and base

**Fig 8:**
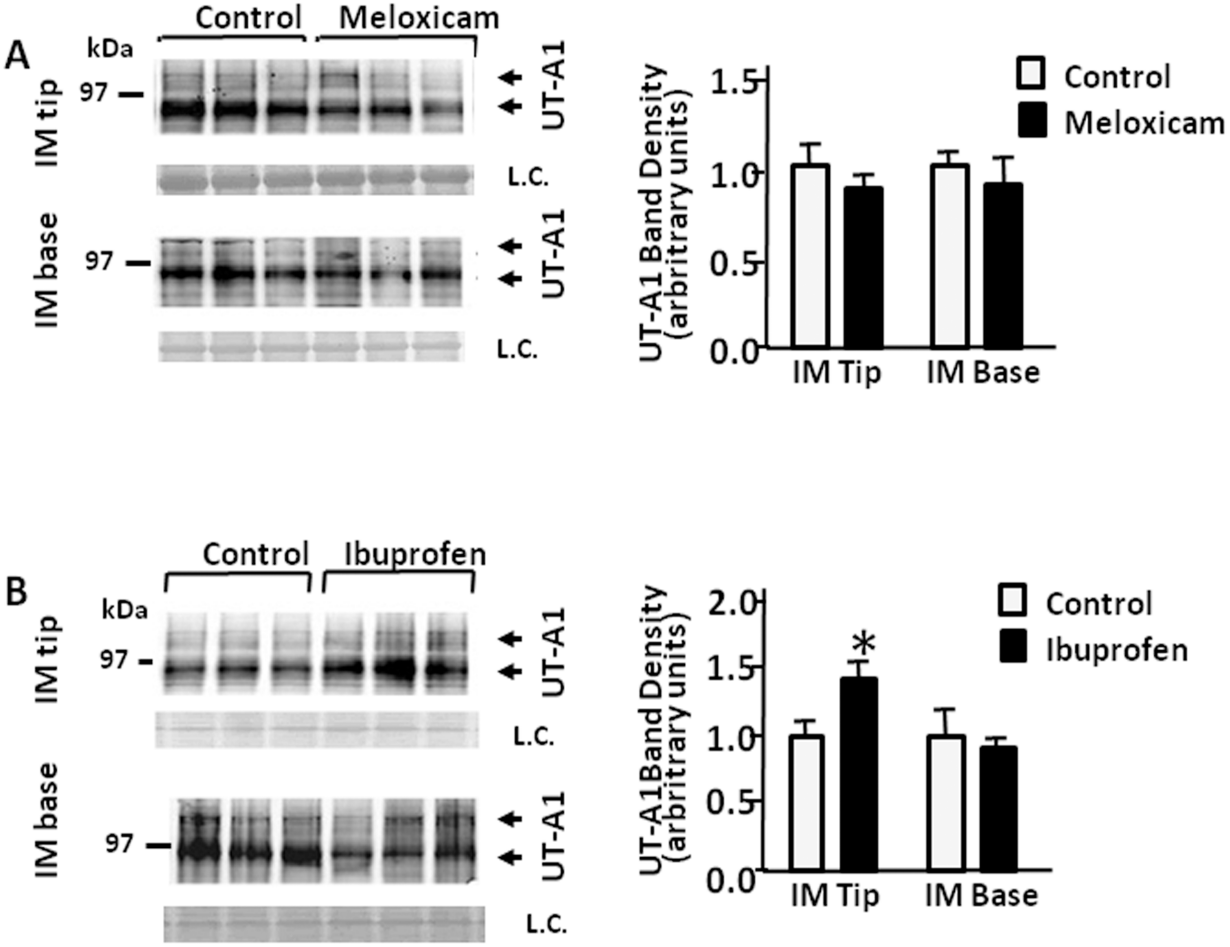
Effect of meloxicam and ibuprofen on UT-A1 abundance in rat inner medullary tip and base. Shown are representative Western blots (left hand panels) from IM tips or bases from rats after 14 days treatment with or without meloxicam (top) or ibuprofen (bottom) probed for total UT-A1 (arrows). The arrows show the two major glycosylated forms of UT-A1 at 117 and 97 kDa. L.C. = loading control. The right hand panels provide combined densitometry from n = 12 rats per group. All proteins were normalized to loading controls. Bars = Mean ± SEM; *P<0.05 compared with control rats.

Total AQP2 was reduced in rat IM tip and base in response to the NSAIDs. We next looked to see if UT-A1 abundance was changed in rat IM tip and base in response to meloxicam or ibuprofen. As shown in Fig 8A, there were no significant differences observed in UT-A1 protein expression in meloxicam-treated rat IM tip or base. However, the abundance of UT-A1 protein was significantly increased in IM tip, but not in IM base of rats treated with ibuprofen (Fig 8B).

## Discussion

NSAID medications are often associated with some adverse effects in renal function. In patients who already suffer from renal dysfunction, chronic use of NSAIDs can lead to hyperkalemia, sodium retention, acute renal failure, reduced glomerular filtration rate, renal papillary necrosis, and edema [6,9]. In patients with normal kidney function and those with renal dysfunction, the prolonged use of NSAIDs may cause a urine concentrating defect [10]. These side effects may be manifested as interstitial fibrosis in the kidney [23]. The acute renal failure related to the use of NSAIDs may be associated with underlying volume depletion, renal insufficiency and some other concomitant diseases [24,25]. Another frequently noted negative side effect is the loss of urine concentrating ability that has been observed in patients [10,26] and has been studied in rodent models [27].

In this study, rats were treated with meloxicam and ibuprofen. These two inhibitors are structurally dissimilar and have different specificities. Meloxicam is a selective inhibitor of COX-2 whereas ibuprofen offers equally effective inhibition of COX-1 or COX-2. Chronic treatment with either of these NSAIDs resulted in increased urine output and decreased urine osmolality 14 days after administration. Serum creatinine and urea were unchanged, suggesting that neither NSAID caused acute kidney injury during this study. Kim et al. reported that rats treated with indomethacin produce a significant decrease in urinary flow rate and decreased urine output [27]. This differs from our results which show a significantly increased urine output and a decreased urine osmolality. Comparison of our results with theirs indicates that NSAIDs have the ability to produce a urine concentrating defect, but the mechanisms might be different, perhaps due to distinct targets.

It has been known for some time that people with impaired kidney function are more prone to experiencing harmful side effects from the chronic use of NSAIDs than people with healthy kidneys. However, people with healthy kidneys can have harmful renal consequences from the chronic use of NSAIDs. Previous studies that examined the mechanisms by which NSAIDs harm already impaired kidneys have mostly used a bilateral ureteral obstruction (BUO) model [28–33]. These studies found that obstruction resulted in decreased levels of both AQP2 and NKCC2. The administration of a COX-2-selective NSAID prevented the decrease in AQP2 levels and partially prevented NKCC2 levels from decreasing. AQP2 levels were not altered in NSAID-treated sham operated control animals. The urine volume was elevated in the bilateral ureteral obstruction (BUO) animals even though the AQP2 levels appeared to be unchanged relative to sham operated control animals [31,32]. Neither phospho-AQP2s nor urea transporters were assessed in these papers.

Our studies were performed on animals with completely normal kidneys and showed both similarities and differences when compared to the effects of NSAIDs on the BUO kidneys. Unlike the sham operated animals in previous studies, our treatment of animals with normal kidneys for 14 days with meloxicam resulted in 36% and 37% decrease in AQP2 levels in IM tip and base, respectively. We saw a similar decrease in the AQP2 level with ibuprofen treatment in IM tip to 70% of normal rats. There was no significant decrease in AQP2 observed in the IM base with ibuprofen treatment. This is in contrast to the BUO model where treatment of animals with impaired kidney function blocked decreases in (but did not increase) AQP2. Although this is an apparent inconsistency, the treatment of the BUO animals with the NSAIDs was for a relatively short duration (3 days) with a different NSAID (parecoxib), as opposed to the current treatment of the normal animals for 14 days with ibuprofen or meloxicam. The explanation may be as simple as the time it takes to establish a new set point for protein levels.

Vasopressin binding to the V2R results in stimulation of adenylyl cyclase and increased cAMP. This, in turn, stimulates protein kinase A (PKA) to phosphorylate AQP2 and UT-A1. Subcellular localization and water permeability of AQP2 are regulated by phosphorylation [34,35]. Specifically, AQP2 is inserted in vesicles, then is translocated to the plasma membrane, as a result of the binding of vasopressin to V2R [36]. Star et al. showed that increases in vasopressin in the IMCD are associated with a transient rise in intracellular calcium [37]. We speculate that this increase in calcium could possibly decrease AQP2 levels while the increase in cAMP accumulation increases AQP2 phosphorylation, but future studies would be needed to test this possibility.

In the present studies, we found that NSAIDs not only reduce total AQP2 expression level, but also increase the p-AQP2 abundance in IM tip and base. Hoffert et al. reported that a rapid increase in p^256^-AQP2 is necessary for subsequent phosphorylation at Ser 264 and Ser 269 in cAMP-mediated regulation of the AQP2 trafficking pathway. P^261^-AQP2 shows a slow decrease in phosphorylation upon exposure to vasopressin [38,39]. Nejsum et al. reported that prostaglandins inhibit cAMP-induced water permeability in rat IMCD and counteracts PKA-activated AQP2 translocation to the apical plasma membrane. This effect is dependent on the phosphorylation of AQP2 at S256. However, in response to prostaglandins, cAMP/PKA-dependent internalization of AQP2 is independent of dephosphorylation of S256 [40]. Phosphorylation of these four serines is considered of vital importance in regulating the subcellular distribution of AQP2 [41].

In our studies, NSAID treatment altered AQP2 phosphorylation at all four sites. The increases in the ratios of p^256^-AQP2, p^264^-AQP2 and p^269^-AQP2 to total AQP2 happened in response to meloxicam and ibuprofen in IM tip. P^261^-AQP2 expression was reduced by meloxicam in IM base and by ibuprofen in IM tip and base. We speculate that the increased phosphorylation of AQP2 in response to NSAIDs may be an attempt to alter the membrane accumulation as a compensatory mechanism for total AQP2 protein loss, thus ultimately increasing water reabsorption to partially correct the polyuric response to the NSAIDs. However, we did find different effects on p-AQP2 with different NSAID treatments. This was particularly apparent in the response of Ser261 phosphorylation to meloxicam as opposed to ibuprofen. To the best of our knowledge, this work is the first description of the effect of NSAIDs on the relative abundances of these four phosphorylated AQP2s in the IM tip versus base.

Both UT-A1 and AQP2 are expressed in the inner medullary collecting duct. Functional deletion of UT-A1 in mice causes significant polyuria and a urine concentration defect [42–44]. To better understand the mechanisms behind the NSAID-mediated urine concentrating defect, we also examined UT-A1 abundance. Although AQP2 decreased in rat IM tip and base, ibuprofen increased UT-A1 expression level in IM tip. This is a common response for UT-A1. While absence of UT-A1 is characterized by a concentrating defect, when UT-A1 is present, it is generally increased in polyuric conditions in an attempt to move more urea into the interstitium to increase the hypertonicity and promote water reabsorption.

In conclusion, our study demonstrates that NSAIDs markedly reduce the expression of AQP2 water channels in the IM tip and base. On the other hand, the phosphorylation of AQP2 increases in response to NSAIDs. We speculate that this may alter the membrane accumulation of AQP2 as a compensatory mechanism, thus potentially enhancing water reabsorption. Future studies will be needed to further elucidate the mechanisms by which NSAIDs increase AQP2 phosphorylation.
